# Triple monoclonal protein-related kidney lesions in a patient with plasma cell dyscrasia: a case report

**DOI:** 10.3389/fneph.2024.1399977

**Published:** 2024-11-08

**Authors:** Arsalan Alvi, Alexander J. Gallan, Nattawat Klomjit

**Affiliations:** ^1^ Division of Nephrology and Hypertension, Department of Medicine, University of Minnesota, Minneapolis, MN, United States; ^2^ Department of Pathology and Laboratory Medicine, Medical College of Wisconsin, Milwaukee, WI, United States

**Keywords:** monoclonal gammopathy of renal significance, MGRS, multiple myeloma, thrombotic microangiopathy, TMA, light chain proximal tubulopathy, LCPT, light chain cast nephropathy

## Abstract

A toxic monoclonal protein typically results in a single kidney pathology due to the specific biophysical characteristics of monoclonal proteins. Multiple monoclonal protein lesions are rarely reported and often portend a poor prognosis. We present a 57-year-old male who developed rapidly progressive glomerulonephritis after concealed ruptured diverticulitis. A kidney biopsy showed light chain cast nephropathy, light chain proximal tubulopathy, and thrombotic microangiopathy. Laboratories showed IgG kappa with an M-spike of 0.2 g/dl and a kappa light chain of 16 mg/dl. A bone marrow biopsy showed 3% kappa-restricted plasma cells. The dramatic renal presentation despite the minimal hematological burden is suggestive of a highly toxic light chain, which is consistent with monoclonal gammopathy of renal significance (MGRS). Clone-directed therapy and a complement blockade were initiated. The patient remained dialysis-dependent despite a hematological response. This case highlights the importance of considering the toxic properties of monoclonal proteins in causing kidney diseases. Our case is the first report of an MGRS patient with three distinct kidney lesions. Triple monoclonal protein-related kidney lesions are very rare and are usually associated with multiple myeloma. Light chain cast nephropathy (LCCN) is a myeloma-defining event but his light chain (LC) (<50 mg/dl) and plasma cell (<10%) burdens were low which makes this case very unusual. Sepsis-induced low-flow stage and the toxic properties of LC may induce LCCN in this patient. Aggressive therapy is likely needed to eradicate the clone in order to achieve an organ response.

## Introduction

Multiple myeloma (MM) is a plasma cell disorder that often affects the kidneys. MM requires the presence of ≥ 10% of monoclonal plasma cells in the bone marrow, or a verified plasmacytoma located in the bone or outside the bone marrow, accompanied by a myeloma-defining event: hypercalcemia, renal insufficiency, anemia, or osteolytic lesions (1). Recently, any presence of imminent myeloma including a free light chain (LC) ratio surpassing 100, bone marrow plasma cells exceeding 60%, or the existence of at least one bony lesion on magnetic resonance imaging (MRI) has also been incorporated in the diagnosis of MM (1). Patients without any organ damage or myeloma-defining events are categorized as monoclonal gammopathy of undetermined significance (MGUS) or smoldering myeloma (SMM) depending on the percentage of plasma cells in the bone marrow (2). Certain MGUS and SMM patients may harbor nephrotoxic monoclonal proteins that cause kidney lesions despite an absence of myeloma-defining events. The term monoclonal gammopathy of renal significance (MGRS) was subsequently coined to describe these patients (3). MGRS lesions include, but are not limited to, immunoglobulin light chain amyloidosis (AL), monoclonal immunoglobulin deposition disease (MIDD), light chain proximal tubulopathy (LCPT), proliferative glomerulonephritis with monoclonal immunoglobulin deposits (PGNMID), monoclonal C3 glomerulopathy [C3 glomerulonephritis (C3GN) and dense deposit disease (DDD)], and thrombotic microangiopathy (TMA) (3). Traditionally, light chain cast nephropathy (LCCN) is classified as a myeloma-defining event; however, LCCN may occur in other conditions such as chronic lymphocytic leukemia (CLL) and Waldenstrom macroglobulinemia (WM) (4, 5). Toxic monoclonal proteins usually result in a single type of kidney pathology due to their specific biophysical properties (6). However, multiple types of lesions are rarely reported and often confer a poorer prognosis (7, 8). Most cases with concomitant monoclonal protein lesions have MM (8). We hereby report an unusual presentation of an MGRS patient who presented with triple monoclonal lesions including LCCN, LCPT, and TMA.

## Case description

**Figure 1:**
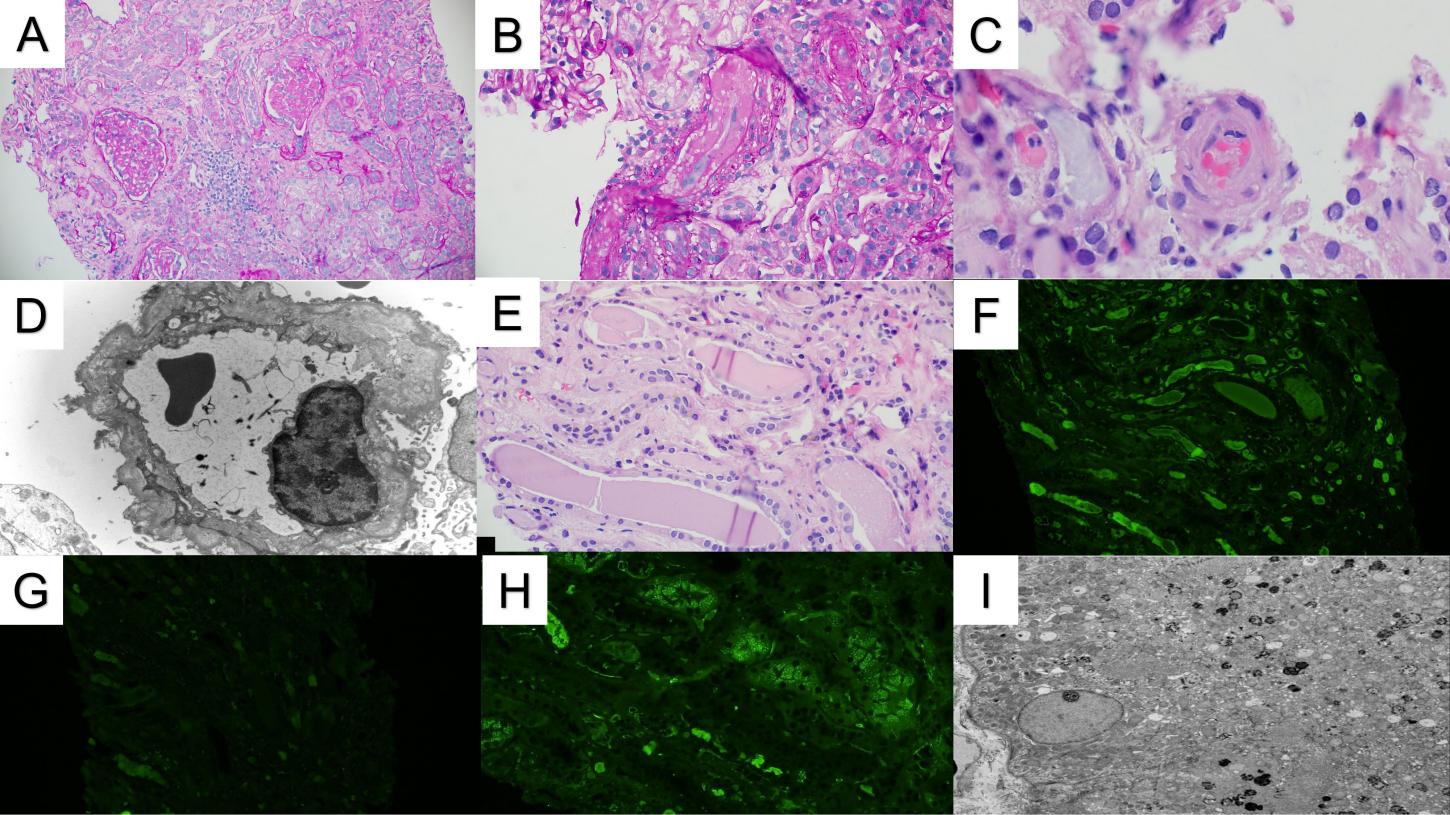
Renal pathology. **(A)** The renal cortex demonstrates patchy glomerular and tubulointerstitial scarring (PAS stain 10x). **(B)** Arterioles show luminal occlusion, endothelial cell swelling, and intimal edema, consistent with microangiopathic injury (PAS stain 20x). **(C)** A few arteriolar intraluminal thrombi are also present (H&E stain, 40x). **(D)** Electron microscopy demonstrated multilayering of the glomerular basement membranes, consistent with chronic microangiopathic injury (10000x). **(E)** Several atypical-appearing tubular casts are present with fragmentation/fracturing (H&E stain 20x). **(F, G)** The atypical casts showed strong immunofluorescence staining for kappa light chains **(F)** without significant staining for lambda light chains **(G)**. **(H)** Kappa light chain immunofluorescence also shows strong tubular reabsorption droplet staining. Similar staining was not observed for lambda light chains (not shown). **(I)** An ultrastructural evaluation of the proximal tubules demonstrated mottled lysosomes, supporting the diagnosis of light chain proximal tubulopathy. Intraepithelial crystals were not observed (2500x).

A 57-year-old white male with a history of dermatomyositis and sarcoidosis on mycophenolate mofetil and prednisone presented with fever, nausea, and loose stools for 3 days. Upon presentation, he was febrile with a temperature of 102°F, blood pressure of 170/80 mmHg, and heart rate (HR) of 60 bpm. The heart and lung examinations were unremarkable. An abdominal examination revealed diffuse mild tenderness without any rebound tenderness. Ankle edema was absent. Laboratory investigation showed a creatinine (Cr) level of 2.81 mg/dl (baseline Cr 1.47 mg/dl at 3 months prior to presentation) which continued to rise and peaked at 9.29 mg/dl. Serum calcium was 8.2 mg/dl. Urinalysis showed a red blood cell count of 4/hpf and a white blood cell count of 7/hpf without cast. The patient’s urine protein creatinine ratio was 1.33 g/g. Hemoglobin was 11.2 g/dl and platelet 191 x10^3^cell/µl but these continued to decrease (hemoglobin 9.2 g/dl and platelet 121 x10^3^cell/µl) as did the presence of hemolysis markers. Haptoglobin was 26 mg/dl, the lactate dehydrogenase (LDH) was 703 U/l, ADAMTS13 activity was 28%, and a peripheral blood smear showed schistocytes (<5%). A direct antiglobulin test was negative. Imaging revealed a concealed ruptured diverticulitis with a normal kidney appearance. The HIV, hepatitis B virus, hepatitis C virus, and SAR-CoV 2 serologies were all negative. The patient’s blood culture was also negative. The patient underwent an urgent kidney biopsy which showed 31% globally sclerotic glomeruli and 10%-15% interstitial fibrosis and tubular atrophy. The non-sclerotic glomeruli demonstrated an ischemic wrinkling of the glomerular basement membrane (GBM) with endothelial cell swelling and rare red blood cell fragments in the glomerular capillaries. There were occasional fragmented and granulated intraluminal casts with a periodic-acid Schiff (PAS)-positive appearance. There was evidence of acute tubular injury with a scattered area of more prominent cytoplasmic protein droplet accumulation without crystallization. The arteries and arterioles showed endothelial cell swelling, focal intimal edema, thrombus formation with onion skin-type changes, and luminal narrowing. Frozen immunofluorescence (IF) revealed no significant immunoreactant staining in the glomeruli. However, pronase-digested paraffin IF revealed kappa light chain restriction in intraluminal casts. Proximal tubules were positive for kappa light chains in intracytoplasmic droplets. Electron microscopy showed endothelial swelling with ischemic wrinkling and focal multi-layering of the glomerular basement membrane. Furthermore, an immune complex was absent. Some proximal tubules showed enlarged, mottled lysosomes within the cytoplasm without distinct crystal formation (**Figure 1**). Given there is no standardized quantification of light chain cast, we did not systemically count these casts. Overall, these findings were consistent with kappa-restricted LCCN, non-crystalline LCPT, and TMA. A subsequent work-up showed a kappa LC of 16 mg/dl, lambda LC of 2.13 mg/dl, and a kappa/lambda (K/L) ratio of 7.17. Serum protein electrophoresis (SPEP) showed IgG kappa with an M-spike of 0.2 g/dl, and a bone marrow biopsy showed less than 3% kappa-restricted plasma cells. Additional work-up showed elevated beta-2 microglobulin at 19.5 mg/l (normal <2.3 mg/l) and negative cryoglobulin. Bone survey and positron emission tomography-computed tomography (PET-CT) scans showed no bony lesions. Fluorescence *in situ* hybridization (FISH) showed no high-risk genetic abnormality with no loss of 1p, 13q, and TP53. There were no gains of chromosomes 5, 9, 15, and 1q. However, the patient had IgH rearrangement (16.5%). Due to the TMA, a complement study was conducted which showed an elevated sC5b-9 without other abnormalities (**Table 1**). Complement gene abnormality was absent for complement factor H (CFH), CFI, CFB, membrane cofactor protein (MCP), CFH-related peptide 5 (CFHR5), and no deletion or duplication on CFH-CFHR5 genes. Given the finding of progressive TMA, the patient underwent plasma pheresis for 3 sessions as a bridging therapy before initiating anti-plasma cell therapy with daratumumab, bortezomib, and dexamethasone (Dara-Vd). Due to a recently ruptured diverticulitis, cyclophosphamide and complement blockade were not initiated in the first cycle. He was started on acyclovir and pentamidine inhalation for antiviral and antibacterial prophylaxis. The patient’s TMA parameters were partially improved (normal haptoglobin and improved LDH at 363 U/l) 7 weeks after chemotherapy initiation. The surgeon and infectious disease doctors agreed to add a complement blockade, and ravulizumab was initiated. He received a meningococcal vaccine that covered both ACWY and B strains 6 weeks prior to the initiation of ravulizumab. Four months after chemotherapy initiation (4 cycles of Dara-Vd), he achieved a very good partial response (VGPR) (9) with kappa LC of 1.70 mg/dl, lambda LC of 1.47, K/L ratio of 1.16, and dFLC of 0.23 mg/dl. SPEP showed monoclonal IgG kappa with an M-spike of 0.1 g/dl. Hemolysis markers significantly improved with haptoglobin of 242 mg/dl and LDH of 294 U/l. Hemoglobin and platelet levels improved to 10.8 g/dl and 204 x10^3^cell/µl, respectively. The treatment course was complicated by neutropenia, a recurrent *Clostridium difficile* infection, and hypogammaglobulinemia. Despite significant improvement in the patient’s monoclonal protein and hemolytic parameters, he did not achieve any meaningful kidney recovery and remained dialysis dependent. Hence, the decision was made to discontinue clone-directed therapy and ravulizumab 4 months after the diagnosis. The patient was disappointed due to his non-recovered kidney function and being dialysis dependent. However, he was able to tolerate dialysis well. At 6 months after stopping the treatment, he still remained in VGPR (9) with kappa LC of 0.68, lambda LC of 0.65, K/L ratio of 1.05, and dFLC of 0.03 mg/dl. SPEP still displayed IgG kappa with 0.1 g/dl spike and his TMA parameters had normalized.

**Table 1:** Complement functional panel upon presentation in this patient.

*Complement functional panel*	*Value*
*Total complement (30-75 U/ml)*	64 U/ml
*Alternative complement pathway function (≥ 46%)*	68%
*Complement C3 (75-175 mg/dl)*	88 mg/dl
*Complement C4 (14-40 mg/dl)*	16 mg/dl
*Complement factor B antigen (15.2-42.3 mg/dl)*	24.3 mg/dl
*Complement factor H antigen (18.5-40.8 mg/dl)*	20.2 mg/dl
*CBb complement (<1.7 mcg/ml)*	1.4 mcg/ml
*sC5b-9 complement (<251 ng/ml)*	369 ng/ml

## Discussion

We present a compelling case that involves a unique scenario in which our patient with MGUS exhibited three distinct monoclonal protein-related kidney lesions. These three lesions included LCCN, LCPT, and TMA. Although the presence of LCCN is a myeloma-defining event, LCCN may occur in other conditions such as chronic lymphocytic leukemia and Waldenstrom’s macroglobulinemia (4, 5, 10). Our patient had a minimal monoclonal protein (M-spike < 3 g/dl) and plasma cell burden (plasma cell <10%) which is consistent with MGUS (2). Moreover, other work-ups for multiple myeloma did not reveal any other findings such as an osteolytic lesion, hypercalcemia, or high-risk genetic abnormalities in the FISH analysis. Recently, a multicenter study of LCCN reported four cases of LCCN in the setting of MGRS (11). In summary, a plasma cell disorder in this patient behaved more like MGRS than typical MM. LCCN in non-MM conditions tends to have a lower light chain burden in the low-flow state setting (hypotension, volume depletion, or sepsis) (4). The cast burden is also minimal and less likely to have a significant impact on the severity of acute kidney injury (AKI), similar to our case. This patient had a slight increase in kappa LC at only 16 mg/dl which is much lower than most LCCN cases. It is likely that sepsis and diverticulitis in our patient enabled the low-flow state and thus precipitated LCCN. It is also possible that the light chain properties of our patient were very toxic and prone to bind to Tamm-Horsfall proteins (THPs), resulting in the formation of a light chain cast. LCCN is rare when light chains are less than 50 mg/dl but the risk increases significantly when concentrations are greater than 80-200 mg/dl (12). However, from large multi-center studies, the median level of light chains was typically much higher upon presentation, ranging from 500-750 mg/dl (11, 13). Risk factors for LCCN include high light chain burden, nephrotoxic agents, volume depletion, and hyperviscosity syndrome (14). Moreover, a higher involved light chain level and lower level of hemoglobin are associated with a requirement for dialysis (11).

Intriguingly, our patient also exhibited a rare form of MGRS, LCPT, which occurs in fewer than 5% of biopsy cases with monoclonal protein-related kidney injury (15). LCPT causes renal damage through intracytoplasmic accumulation of toxic light chains, which in turn leads to tubular injury. The pathogenesis is believed to result from insufficient lysosomal degradation of toxic light chains, leading to intracellular accumulation and crystallization (16). Most LCPT cases have kappa light chains and approximately 90% of cases have the crystalline type (6, 16, 17). Notably, our patient had a rare form of non-crystalline type LCPT which comprises only 10% of all LCPT cases (17). In the non-crystalline type, the lysosome containing the light chain bursts, which releases hydrolytic enzymes and leads to cytoplasmic vacuolization and cellular necrosis (16). Approximately two-thirds of LCPT have either incomplete or complete Fanconi syndrome, which includes hypokalemia, aminoaciduria, phosphaturia, glycosuria with normal serum glucose, and proximal renal tubular acidosis (17). Our patient did not have Fanconi syndrome and one plausible explanation for this was minimal urine output and limited renal clearance of electrolytes and glucose.

Another noteworthy pathological finding in our case was the presence of TMA, a condition characterized by evidence of endothelial injury with or without the formation of thrombi in arterioles and capillaries (18). The underlying mechanism of monoclonal protein-associated TMA is believed to be two-fold. First, it may occur through direct endothelial injury from monoclonal proteins which activates the cytokines that trigger the injurious cascade which leads to TMA (19, 20). Alternatively, monoclonal proteins can act as auto-antibodies to complement regulatory proteins such as factor H, resulting in an upregulation of the alternative complement pathway, contributing to the development of TMA (19, 21). The patient’s complement functional assay showed no abnormalities except a mildly elevated terminal complement complex (sC5b-9). Genetic analysis was also negative for a complement gene abnormality. This may suggest the mechanism of TMA in our patient was mostly direct endothelial injury, which is consistent with a recent study of monoclonal-mediated TMA (20).

In our patient’s case, even though the percentage of monoclonal cells was less than 10%, it was crucial to pursue aggressive treatment, especially considering the rapid deterioration of kidney function and the patient’s reliance on dialysis. Therefore, clone-directed therapy was pursued. Plasma pheresis was initially performed primarily to aid in the removal of toxic light chains while waiting for the initiation of chemotherapy due to an active infection. Although we realized that the benefit of plasmapheresis in LCCN is controversial (12, 22), we decided to offer plasmapheresis due to the severe renal presentation. Moreover, plasmapheresis may also be beneficial in certain TMA cases (23). The patient’s TMA initially had a partial response after clone-directed therapy, suggesting that reducing light chains could partially ameliorate TMA. A complement blockade was subsequently added at 7 weeks post-chemotherapy to further improve TMA and kidney response. Despite this, he did not have any meaningful kidney recovery. It is possible that the presence of TMA in our case contributed to his poor renal prognosis since the renal or survival outcomes of patients with monoclonal protein-related TMA (24) seem to be inferior compared to other types of MGRS such as LCPT, PGNMID, and C3GN despite clone-directed therapy (17, 25, 26). Moreover, patients with multiple monoclonal protein-related kidney lesions also have poorer renal and survival outcomes (8, 10).

Our case highlights the toxic properties of monoclonal proteins despite the minimal hematological burden. While toxic monoclonal proteins typically cause single renal pathology, some may incite multiple types of injury (6). Approximately a third of MIDD and fewer than 5% of LCPT patients may have concomitant LCCN (7, 27). MIDD may also present in 1% of AL amyloidosis (8). However, these cases usually present in the setting of multiple myeloma (7, 8, 27). Therefore, it is rare for multiple monoclonal protein-related kidney lesions to occur in MGRS. The pathogenesis of concomitant kidney lesions remains unclear but could be due to the presence of more than one pathological plasma cell subclone, resulting in different conformational structures of the same nephrotoxic monoclonal protein (8). However, we could not test whether the patient had any subclones. We also did not analyze the variable regions of the patient’s monoclonal proteins to gain insight into the properties of this light chain since the difference in variable regions of monoclonal proteins may be associated with the nephrotoxic properties of the light chains (28).

In summary, our case is the first report of MGRS with three distinct monoclonal protein-related kidney lesions. While LCCN is a myeloma-defining event, it may occur in MGRS, especially in the low-flow state setting, similar to our patient. Despite initiating clone-directed therapy and achieving complement blockade with a good hematological response, there was no renal response, which may be due to the property of toxic light chains and degree of renal injury.

## Data Availability

The raw data supporting the conclusions of this article will be made available by the authors, without undue reservation.
